# ELK1 Enhances Pancreatic Cancer Progression Via LGMN and Correlates with Poor Prognosis

**DOI:** 10.3389/fmolb.2021.764900

**Published:** 2021-12-13

**Authors:** Qiang Yan, Chenming Ni, Yingying Lin, Xu Sun, Zhenhua Shen, Minjie Zhang, Shuwen Han, Jiemin Shi, Jing Mao, Zhe Yang, Weilin Wang

**Affiliations:** ^1^ Department of General Surgery, Huzhou Central Hospital, Huzhou, China; ^2^ Department of Pancreatic Hepatobiliary Surgery, Changhai Hospital, Shanghai, China; ^3^ Department of Neurosurgery, RenJi Hospital, Shanghai JiaoTong University School of Medicine, Shanghai, China; ^4^ Department of Hepatobiliary and Pancreatic Surgery, Department of Liver Transplantation, Shulan (Hangzhou) Hospital, Zhejiang Shuren University School of Medicine, Hangzhou, China; ^5^ Department of Hepatobiliary and Pancreatic Surgery, The Second Affiliated Hospital of Zhejiang University, School of Medicine, Hangzhou, China

**Keywords:** pancreatic carcinoma, LGMN, Elk1, proliferation, invasion, apoptosis

## Abstract

Pancreatic cancer is one of the most lethal cancers and its prognosis is extremely poor. Clarification of molecular mechanisms and identification of prognostic biomarkers are urgently needed. Though we previously found that LGMN was involved in pancreatic carcinoma progression, the upstream regulation of LGMN remains unknown. We used reliable software to search for the potential transcription factors that may be related with LGMN transcription, we found that ELK1 could be a new regulator of LGMN transcription that binded directly to the LGMN promoter. Moreover, knocking down of ELK1 reduced pancreatic cancer cells proliferation, invasion and survival, while LGMN restored the malignancy of pancreatic cancer *in vitro* and *in vivo*. Overexpression of ELK1 further increased cancer cells proliferation, invasion and survival. Clinically, ELK1 and LGMN were positively correlated with clinical stage, degree of differentiation and Lymph node infiltration. ELK1 and LGMN were identified as independent prognostic factors for overall survival. The patients with low expression of ELK1/LGMN survived an average of 29.65 months, whereas those with high expression of ELK1/LGMN survived an average of 16.67 months. In conclusive, our results revealed a new mechanism by which ELK1 promoted the progression of pancreatic cancer *via* LGMN and conferred poor prognosis.

## Introduction

Pancreatic adenocarcinoma is one of the most common malignancies (Bray et al., 2018). Due to the symptoms of pancreatic adenocarcinoma are usually non-specific, the early diagnosis rate is extremely low, which results in the late detection of pancreatic adenocarcinoma, with extensive metastasis and poor prognosis ((Wood, 2014), (Liu et al., 2015)). The median survival time of patients with locally advanced pancreatic cancer was merely 8–12 months. The medium survival time of metastatic cancer patients was only 3–6 months (Colbert et al., 2014). The 5-year survival rate for patients with metastatic pancreatic ductal adenocarcinoma (PDAC) is only 8% (the lowest survival rate of all types of cancer), and pancreatic adenocarcinoma is the fourth leading cause of cancer-related death ((Jagadeeshan et al., 2015), (Ibrahim and Wang, 2016)). Despite the introduction of new therapies, the survival rates of PDAC patients has not increased significantly in recent years (Al Haddad and Adrian, 2014). Thus, there is an urgent need to identify potential mechanisms for pancreatic cancer metastasis.

Legumain (LGMN), also calls asparaginyl endopeptidase (AEP), belongs to the C13 family of cysteine proteases. LGMN specifically cleaves peptide bonds in asparaginyl residue in the mammalian genome (Haugen et al., 2013). Normally, LGMN exists in acidic endosomes/lysosomes and participates in intracellular protein degradation under physiological conditions (Herskowitz et al., 2012). LGMN functions in kidney physiology (Miller et al., 2011), immunity (van Endert, 2009) and osteoclast formation (Choi et al., 1999). LGMN has been determined to be highly expressed in many solid tumors, including colorectal cancer, breast cancer and glioblastoma (GBM), and high expression of LGMN correlated with a more metastatic phenotype, which is partially mediated by the activation of cathepsins and pro-MMP2 (Zhen et al., 2015; D’Costa et al., 2014; Sevenich and Joyce, 2014; Edgington-Mitchell et al., 2015). Recently, LGMN has been shown to exhibit a vesicular staining pattern, and high expression of LGMN was significantly related to an advanced tumor stage, a high Gleason score, perineural invasion, and large pancreatic adenocarcinoma tumors (Zhu et al., 2016). Although we previously found that high LGMN expression was involved in the progression of pancreatic carcinoma in an exosome-dependent manner and LGMN could independently indicate poor prognosis, the upstream regulation of LGMN remains unknown (Yan et al., 2018).

In this study, we investigated the transcriptional regulation of LGMN in pancreatic cancer cells and analyzed the functional interaction between the transcription factors and LGMN *in vitro* and *in vivo*. Their correlation was also analyzed in clinical samples.

## Materials and Methods

### Patients and Tissue Samples

Our study was approved by the Ethics Committee of Huzhou Central Hospital (Approval number 20141103-01, Date of approval: November 27, 2014). Written consent was obtained from patients enrolled in this study. A total of 176 patients (males: 108, females: 68) with histologically confirmed PDAC at Shanghai Changhai Hospital were recruited for this study. The mean patient age was 60.6 years (range 32–75). Patient diagnoses were independently reviewed by two pathologists and classified by WHO criteria. Follow-up data were completed on May 1, 2019.

### Cell Lines

The human pancreatic cancer cell lines PANC-1, BxPC3, SW1990 and ASPC-1 were purchased from the Cell Bank of the Chinese Academy of Sciences. BxPC3 and ASPC-1 were cultured in RPMI1640 medium. SW1990 was cultured in Leibovitz’s L-15 Medium. PANC-1was maintained in Dulbecco’s Modified Eagle Medium (DMEM). All the medium were supplemented with 10% heat-inactivated fetal bovine serum (FBS; Invitrogen, Carlsbad, CA, United States), penicillin (100 U/ml), and streptomycin (100 μg/ml) at 37°C in a humidified atmosphere of 5% CO_2_. All the cells were free of mycoplasma contamination.

### Plasmids and Reagents

Lentivirus vectors for ELK1 and LGMN knockdown or overexpression were constructed by Hanyin Biotech (Hanyin Biotech, Shanghai, China). The lentivirus was packaged using psPAX2 and pMD2G (Hanyin Biotech, Shanghai, China). To obtain stable cells with ELK1 or LGMN knockdown or overexpression, lentivirus supernatant was added to cells, followed by selection with 1 μg/ml puromycin for 2 weeks. siRNA sequences for transcription factors are listed in Supplementary Table S1.

Anti-human LGMN antibody (AF2199, MAB2199; R&D), anti-human ELK1 antibody (ab27708; Abcam), anti-actin antibody (ab8227; Abcam), anti-goat IgG (ab6741; Abcam) and anti-rabbit IgG (#7074; Cell Signaling Technology) were used in this study.

### Western Blot Analysis

Lysates (50 μg per lane) collected from pancreatic cancer cells or exosomes were separated by sodium dodecyl sulfate (SDS)-polyacrylamide gel electrophoresis and transferred to nitrocellulose membranes. After incubation with 5% nonfat milk for 30 min at 25°C, the membranes were further incubated with primary antibodies (1:500 dilution) overnight at 4°C. Horseradish peroxidase-conjugated secondary antibodies (1:3,000 dilution) were added and incubated for 60 min at 25°C. Immune-reactive proteins were captured by enhanced chemiluminescence (ECL).

### Total RNA Isolation and Quantitative Real-Time PCR (qRT-PCR) Analysis

Total RNA was extracted from pancreatic adenocarcinoma cancer cells using TRIzol reagent according to the manufacturer’s instructions (Invitrogen). cDNA was transcribed from 1 μg of total RNA. qRT-PCR was performed with SYBR Premix Ex Taq (TaKaRa, Dalian, China). PCR primers are listed in Supplementary Table S2 (Shenggong Biotech, Shanghai, China).

The cycling conditions were as follows: initial denaturation at 95°C for 5 min, followed by 36 cycles of denaturation at 95°C for 10 s and annealing at 60°C for 30 s. The relative mRNA expression levels were calculated using the comparative Ct (ΔΔCt) method. Actin was used as an internal control.

### CCK8 Assay

We examined cell proliferation using CCK8 assays (Dojindo, Japan).

### Cell Invasion Assay

Cells were seeded onto the upper chamber of Matrigel-coated transwell inserts with a pore size of 8 μm in serum-free medium. FBS (10%) was added to the lower chamber as a chemoattractant. After 24 h, the upper surface of the insert was gently scratched with a cotton swab. Cells invading the lower chamber were fixed with 4% paraformaldehyde and stained with crystal violet. The numbers of invading cells were counted under a microscope. Five random microscopic fields were analyzed for each insert.

### Flow Cytometry (FCM) Analysis

Cell apoptosis was analyzed by annexin V staining. Briefly, cells were seeded for 24 h and then transfected with the appropriate lentivirus for another 48 h. The cells were then harvested, washed twice with PBS, stained with annexin V and DAPI in binding buffer, and detected by FCM after a 15-min incubation at room temperature in the dark. Early apoptotic cells (annexin V+/DAPI−) and late apoptotic cells (annexin V+/DAPI+) were quantified.

### Nude Mouse Model

Mouse experiments of our study was approved by the Animal Ethics Committee of Huzhou Institute of Food and Drug Inspection (Approval number HSYJ2017001, Date of approval: December 14, 2017). Male BALB/c athymic nude mice, 4–6 weeks old and weighing 20–22 g, were purchased from the SLAC (Shanghai, China). The following ASPC-1 cells (5 × 10^6^) were subcutaneously injected into mice: (Bray et al., 2018) ASPC-1-NC (negative control), (Wood, 2014) ASPC-1 ELK1-KD (knockdown), and (Liu et al., 2015) ASPC-1 ELK1-KD/LGMN-OE (overexpressing). Tumor volumes were monitored every week. After 6 weeks, all the mice were euthanized, and the tumors were collected.

### Histological and Immunohistochemical Analyses

Histological and immunohistochemical analyses were performed as previously described (Liu et al., 2003). Rabbit anti-ELK1 antibody (ab32106, lot#: GR259320-21; Abcam) and rabbit anti-LGMN antibody (ab232870, lot#: GR97368-48; Abcam), diluted 1:50 in blocking buffer, were used as primary antibodies. Normal rabbit immunoglobulin G (Abcam) was included as a negative control. Three pathologists from Shanghai Changhai Hospital individually scored samples in a blinded manner before drawing conclusions.

### Quantification of IHC Parameters

ELK1 and LGMN were selected as markers respectively. The expression of these immunosuppressive proteins in tissues were evaluated *via* immunohistochemical analysis. Two pathologists to score the intensity and the percentage of positive cells in the tumor tissue independently. Staining intensity: 0 (No staining); 1 (Light yellow); 2 (Light brown); 3 (Brown). The percentage of positive cells: 0 (<5%); 1 (5–25%); 3 (>25–50%); 4 (≥50–75%); 4 (>75%), these two grades were multiplied and specimens were assigned to four groups according to the achieved score: 0–3, negative; 4–6, weak positive (+); 7–9, moderate positive (++); 10–12, strong positive (+++). Negative control staining was carried out with cold PBS in place of primary antibody. Known positive tissues were used as positive controls. Five fields were randomly taken from each sheet and photographed (magnification ×200–×400)

### Statistical Analysis

Survival was calculated starting from the date of surgery to the date of death or the last follow-up. Survival curves for LGMN were plotted using the Kaplan–Meier method and compared using the log-rank test. The median time and hazard ratio are shown with the 95% confidence interval (CI). Data are presented as the mean ± SD or as the number and percentage. The differences between groups and categorical variables were compared by the chi-square test. For normally distributed data, continuous variables were compared *via* an independent samples *t* test. Statistical analysis was performed with SPSS 15.0 (Chicago, IL, United States). Significance was defined as a *p* value <0.05.

## Results

### ELK1 Promoted LGMN Expression in Pancreatic Cancer Cells

**FIGURE 1:**
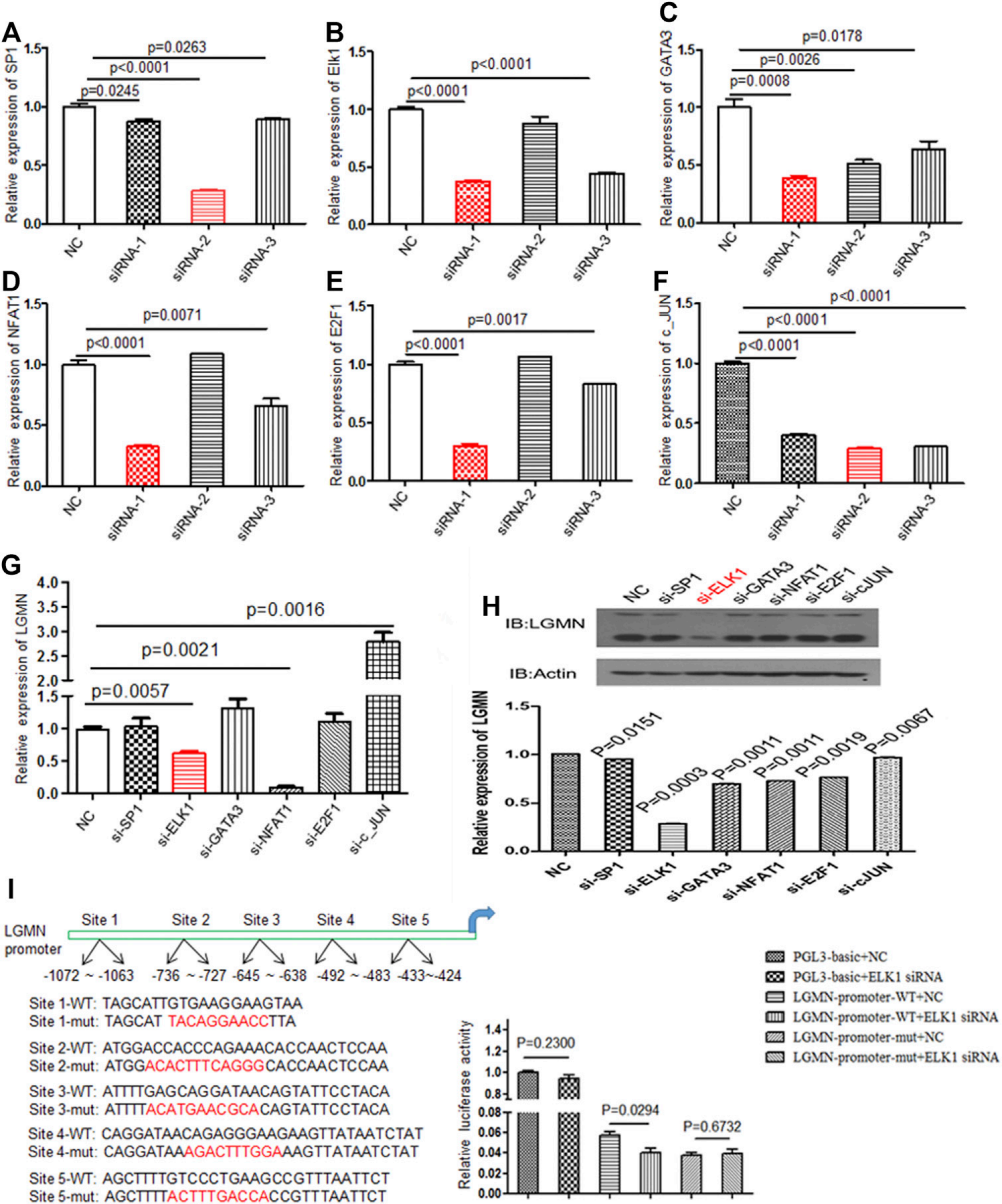
Knockdown of ELK1 reduced LGMN expression in pancreatic cancer cells. **(A**–**F)** RT-PCR analysis of SP1, ELK1, GATA3, NFAT1, E2F1 and c-JUN levels after transfection of respective siRNAs in PANC-1 cells. Three types of siRNA were used. **(G)** RT-PCR analysis of LGMN mRNA expression after transfection of effective siRNAs in PANC-1 cells, the mRNA level was normalized to GAPDH. *p* = 0.0057 between ELK1-KD and control group, *p* = 0.0021 between NFAT1 and control group. **(H)** Western blot analysis of LGMN levels after transfection of effective siRNAs in PANC-1 cells. **(I)** Luciferase assay of LGMN transcriptional activity after transfection of ELK1 siRNAs or control in PANC-1 cells. *p* = 0.029 between LGMN-promoter-WT+siELK1 and control group.

Although we previously found that high expression of LGMN is involved in the progression of PDAC, the upstream regulation of LGMN remains unknown (18). In order to study the upstream regulation of LGMN in PDAC, we used JASPR and PROMP to search for potential transcription factors in LGMN promoter. SP1, ELK1, GATA3, NFAT1, E2F1 and c-JUN scored high in both software programs. Therefore, we knocked down each of these factors by siRNAs (Table 1) and determined their effect on LGMN expression in PANC-1 cells (Figures 1A–F). As shown in the figure, the mRNA level of LGMN decreased significantly after knockdown of ELK1 and NFAT1 (*p = 0.0057, **p = 0.0021, Figure 1G), LGMN protein also decreased after the inhibition of ELK1 (*p = 0.0003, Figure 1H and Supplementary Figure S5). Therefore, we constructed the LGMN promoter, and luciferase detection showed that knockdown of ELK1 significantly reduced the transcriptional activity of LGMN mRNA (*p = 0.029, Figure 1I).

**TABLE 1:** Association of LGMN expression with clinicopathological variables in pancreatic ductal adenocarcinoma.

Clinicopathological characteristic	*n* (%)	LGMN staining (*n*; %)		*p*-value
		Low	High	
Age (year)				0.137
<60	86 (48.86)	42 (48.84)	44 (51.16)	
≥60	90 (51.14)	54 (60.00)	36 (40.00)	
Sex				0.224
Male	108 (61.36)	55 (50.93)	53 (49.07)	
Female	68 (38.64)	41 (60.29)	27 (39.71)	
Clinical stages				*0.004
I/II	138 (78.41)	83 (60.14)	55 (39.86)	
III/IV	38 (21.59)	13 (34.21)	25 (65.79)	
Tumor location				0.198
Head	112 (63.64)	57 (50.89)	55 (49.11)	
Body/tail	64 (36.36)	39 (60.93)	25 (39.07)	
Tumor size				0.160
≤4 cm	58 (32.95)	36 (62.07)	22 (37.93)	
>4 cm	118 (67.05)	60 (50.85)	58 (49.15)	
Differentiation				*0.012
Weak to moderately	110 (62.50)	68 (61.82)	42 (38.18)	
Poorly	66 (37.50)	28 (42.42)	38 (57.58)	
N infiltration				0.060
Positive	165 (93.75)	87 (52.73)	78 (47.27)	
Negative	11 (6.25)	9 (81.82)	2 (18.18)	
Lymphnode infiltration				*0.020
Positive	128 (72.73)	63 (49.22)	65 (50.78)	
Negative	48 (27.27)	33 (68.75)	15 (31.25)	

**FIGURE 2:**
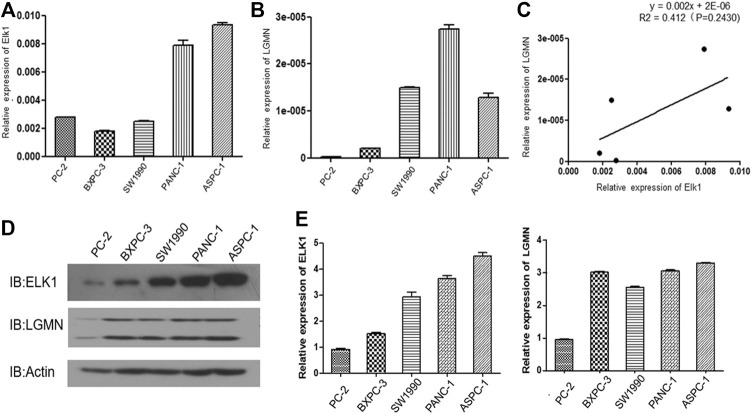
ELK1 positively correlated with LGMN expression in pancreatic cancer cells. **(A**,**B)** ELK1 and LGMN expression in four pancreatic cancer cell lines were detected by RT-PCR. **(C)** Correlation between ELK1and LGMN in pancreatic cancer cells were analyzed. **(D**,**E)** ELK1, LGMN expression and beta-catenin in four pancreatic cancer cell lines determined by Western blot.

**FIGURE 3:**
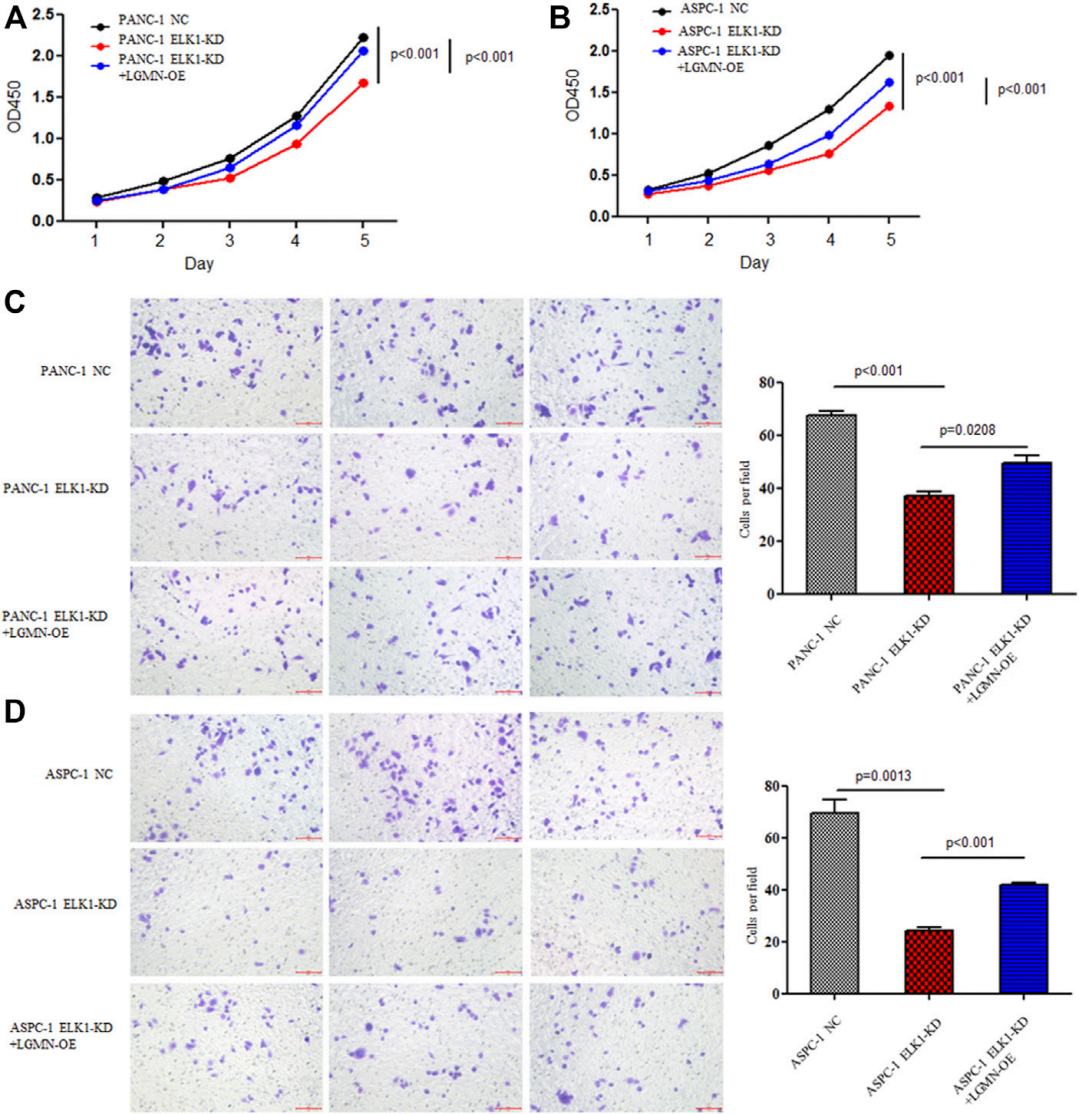
ELK1 promoted pancreatic cancer cell proliferation and invasion *via* LGMN. **(A**,**B)** CCK8 analysis of PANC-1 and ASPC-1 cells with ELK1 knocked down as well as LGMN rescued. *p < 0.001, **p < 0.001, between ELK1-KD group and control group, ELK1-KD group and ELK1-KD+LGMN-OE group in PANC-1 cells **(A)**. *p < 0.001, **p < 0.001, between ELK1-KD group and ELK1-KD+LGMN-OE group in ASPC-1 cells **(B)**. **(C**,**D)** Matrigel-Transwell analysis of PANC-1 and ASPC-1 cells with ELK1 knocked down as well as LGMN rescued. Five random microscopic fields were analyzed for each insert (shown at ×200 magnification).

We then analyzed the expression of ELK1 and LGMN in four different pancreatic cancer cell lines. RT-PCR results showed that ELK1 was relatively high in PANC-1 and ASPC-1 cells compared with BXPC3 and SW1990 cells (Figure 2A), LGMN was relatively high in PANC-1, ASPC-1 and SW1990 cells compared with BXPC3 cells (Figure 2B). A positive correlation between ELK1 and LGMN was observed in the mRNA level of pancreatic cancer cells (*R* ^2^ = 0.412, *p* = 0.2430, Figure 3C).As shown in results, ELK1 protein expression was relatively high in PANC-1 and ASPC-1 cells compared with BXPC3 and SW1990 cells (Figure 2D), LGMN protein expression was higher than that in control (Figure 2D). We chose to overexpress ELK1 in BXPC3 and SW1990 cells and found the protein level of ELK1 increased (Supplementary Figure S2A). We also knocked down ELK1 in PANC-1 and ASPC-1 cells and found the protein level of ELK1 and LGMN decreased, while the expression of LGMN was saved in cells supplemented with over-expressed LGMN (Supplementary Figure S2B). Taken together, these results indicated that transcription factor ELK1 promoted the expression of LGMN in pancreatic cancer cells.

### ELK1 Promoted Pancreatic Cancer Cells Proliferation, Invasion and Survival *via* LGMN

**FIGURE 4:**
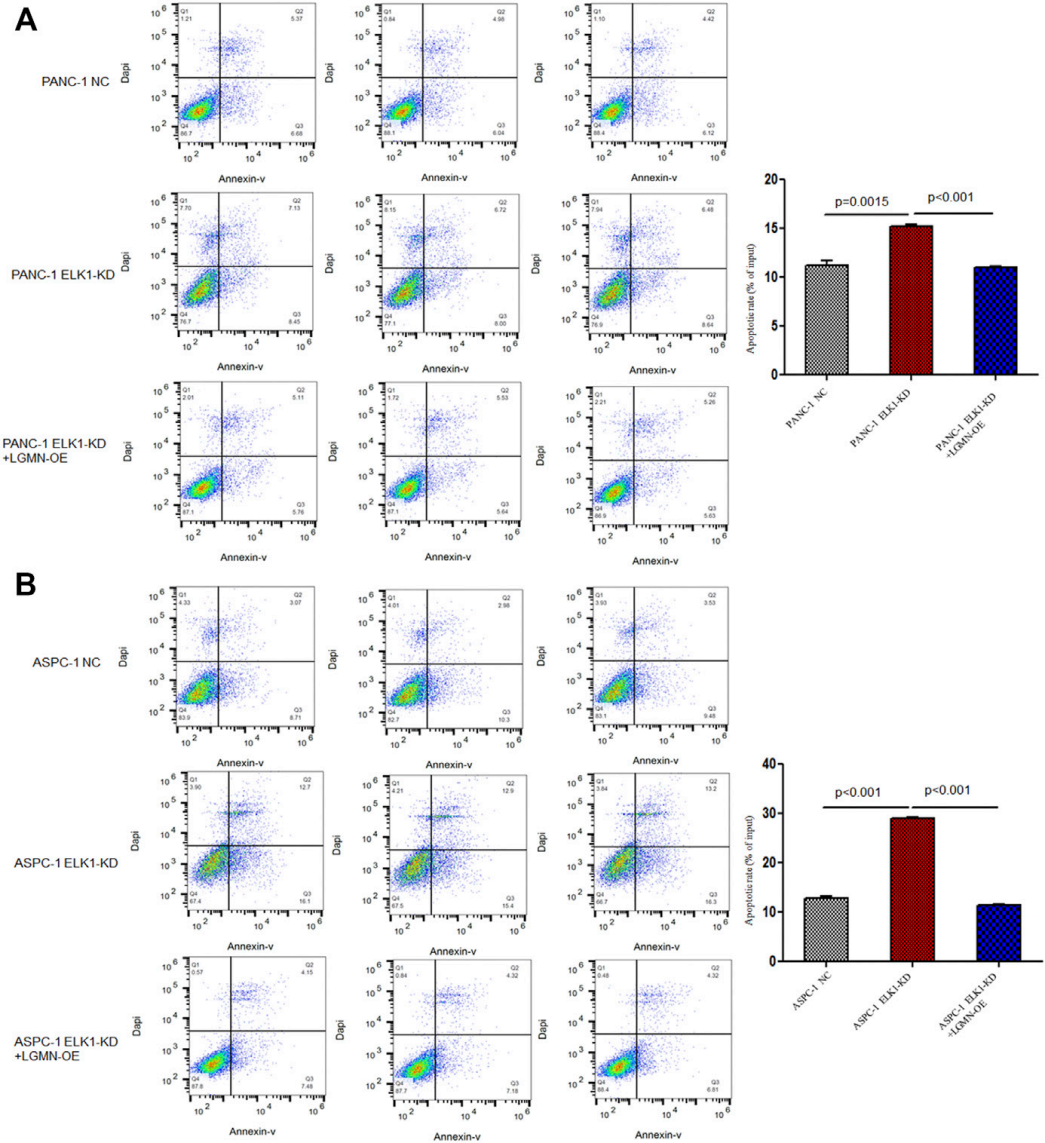
ELK1 promoted pancreatic cancer cells survival *via* LGMN. **(A**,**B)** FACS apoptotic analysis of PANC-1 and ASPC-1 cells with ELK1 knocked down as well as LGMN rescued. *p = 0.0015, **p < 0.001 between ELK1-KD group and control group, ELK1-KD group and ELK1-KD+LGMN-OE group in PANC-1 cells. *p < 0.001, **p < 0.001, between ELK1-KD group and control group, ELK1-KD group and ELK1-KD+LGMN-OE group in ASPC-1 cells.

**FIGURE 5:**
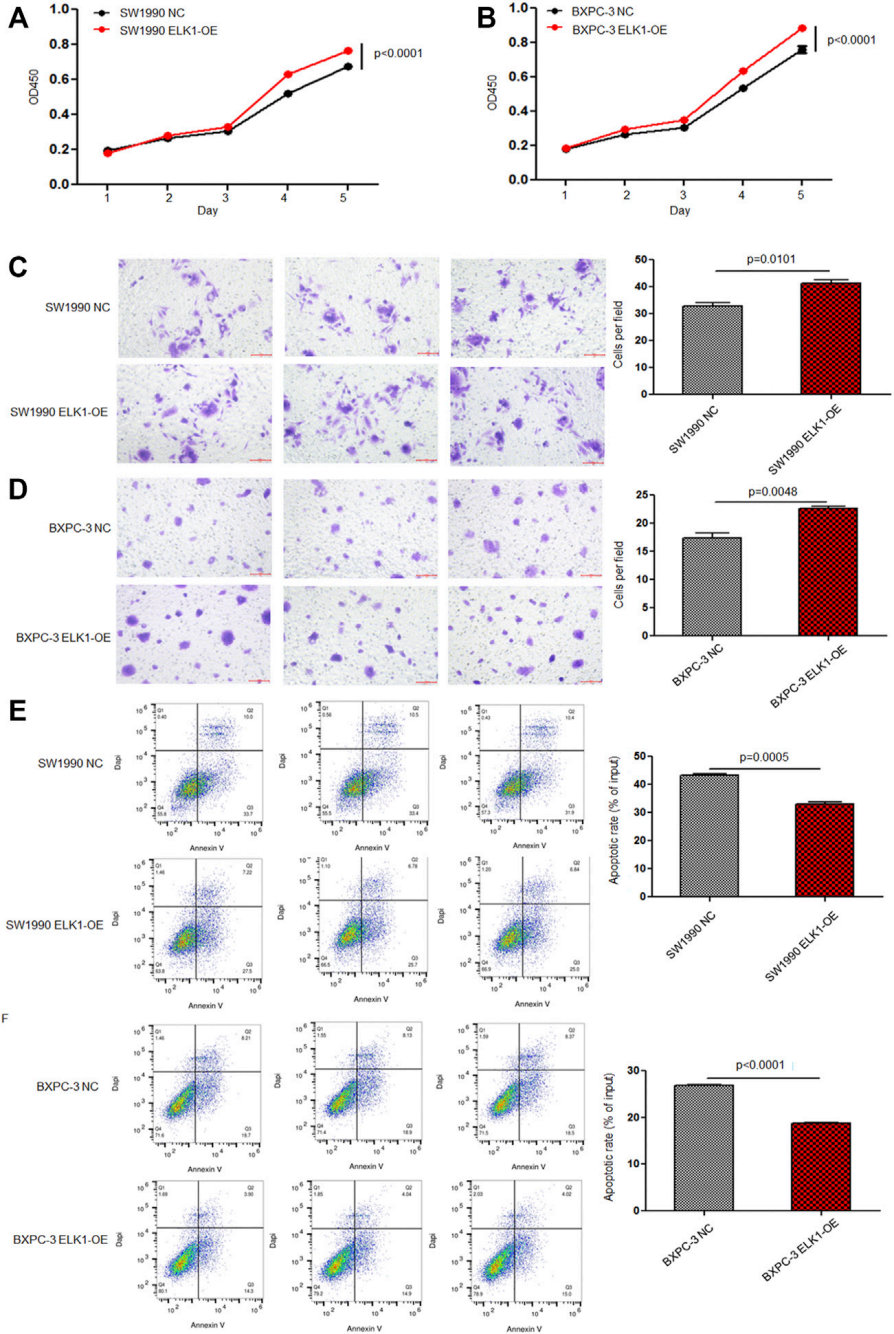
ELK1 overexpression promoted pancreatic cancer cells proliferation, invasion and survival. **(A**,**B)** CCK8 analysis of SW1990 and BxPC3 cells with or without ELK1 overexpression. *p < 0.001, **p < 0.001, between ELK1-OE group and control group in SW1990 and BxPC3 cells. **(C**,**D)** Matrigel-Transwell analysis of SW1990 and BxPC3 cells with or without ELK1 overexpression. *p = 0.0101, **p = 0.0048, between ELK1-OE group and control group in SW1990 and BxPC3 cells. **(E**,**F)** FACS apoptotic analysis of SW1990 and BxPC3 cells with or without ELK1 overexpression. *p = 0.0005, **p < 0.001, between ELK1-OE group and control group in SW1990 and BxPC3 cells.

To elucidate the role of ELK1 in PDAC, we examined the proliferation, invasion and survival of pancreatic cancer cells. CCK8 experiment showed that knockdown of ELK1 gene significantly inhibited the proliferation of PANC-1 and ASPC-1 cells (*p < 0.001, **p < 0.001, Figures 3A,B). In addition, Matrigel Transwell invasion experiment indicated that inhibition of ELK1 weakened the invasive capacity of PANC-1 and ASPC-1 cells (*p < 0.001, **p = 0.0013, Figures 3C,D). Fluorescence-activated cell sorting (FACS) showed that the percentage of apoptotic cells increased after knocking down of ELK1 in PANC-1 and ASPC-1 cells (*p = 0.0015, **p < 0.001, Figures 4A,B), and yet LGMN rescued ELK1-KD cells and restored the proliferation, invasion and survival of PANC-1 and ASPC-1 cells (Figures 3, 4). Furthermore, overexpression of ELK1 promoted proliferation, invasion and survival of SW1990 and BXPC3 cells (Figure 5). The CCK8 assay showed that overexpression of ELK1 gene significantly promoted the proliferation of SW1990 and BXPC3 cells (*p < 0.001, **p < 0.001, Figures 5A,B). Matrigel Transwell invasion experiment indicated that overexpression of ELK1 enhanced the invasive capacity of SW1990 and BXPC3 cells (*p = 0.0101, **p = 0.0048, Figures 5C,D). Fluorescence-activated cell sorting (FACS) showed that the percentage of apoptotic cells decreased after SW1990 and BXPC3 cells overexpressed ELK1 (*p = 0.0005, **p < 0.001, Figures 5E,F). In conclusion, our study revealed that ELK1 promoted the proliferation, invasion and survival of pancreatic cancer cells by LGMN.

**FIGURE 6:**
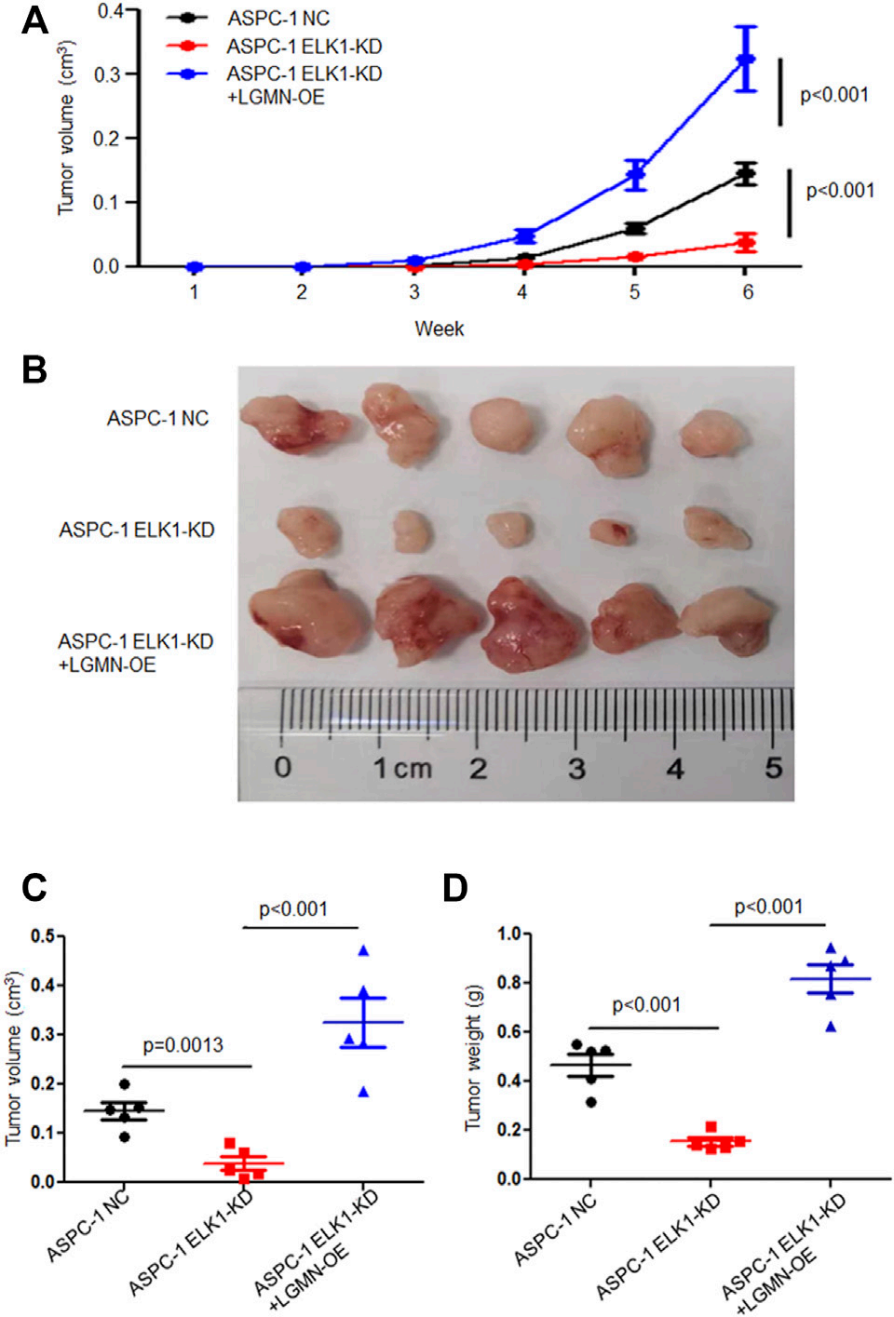
ELK1 promoted pancreatic cancer cells progression *via* LGMN *in vivo*. **(A**–**C)** Tumor volume of ASPC-1 cells with ELK1 knocked down as well as LGMN rescue were monitored. *p < 0.001, **p < 0.001, between ELK1-KD group and control group, ELK1-KD+LGMN-OE group and control group in ASPC-1 cells **(A)**. *p = 0.0013, **p < 0.001, between ELK1-KD group and control group, ELK1-KD group and ELK1-KD+LGMN-OE group in ASPC-1 cells **(C)**. **(D)** Tumor weight of ASPC-1 cells with ELK1 knocked down as well as LGMN rescued were analyzed. *p < 0.001, **p < 0.001, between ELK1-KD group and control group, ELK1-KD group and ELK1-KD+LGMN-OE group in ASPC-1 cells.

To further confirm the effects of ELK1 on PDAC development *in vivo*, we constructed Xenograft models by subcutaneous injection ASPC-1 NC cells, ASPC-1 ELK-KD cells and ASPC-1 ELK1-KD/LGMN-OE cells. Tumor volume analysis showed that tumors grew slower in ELK1-KD group than that in ELK1-KD/LGMN-OE group (*p < 0.001, **p < 0.001, Figures 6A–C). Consistent with the above results, tumors were lighter in ELK1-KD group than that in ELK1 -KD/LGMN-OE group (*p* < 0.001, *p* < 0.001, Figure 6D). Collectively, these *in vivo* experiment confirmed our *in vitro* data and identified ELK1 promoted pancreatic cancer cell proliferation, invasion and survival through LGMN.

### Relationship Between ELK1 and LGMN and Prognosis in Pancreatic Adenocarcinoma Patients

**FIGURE 7:**
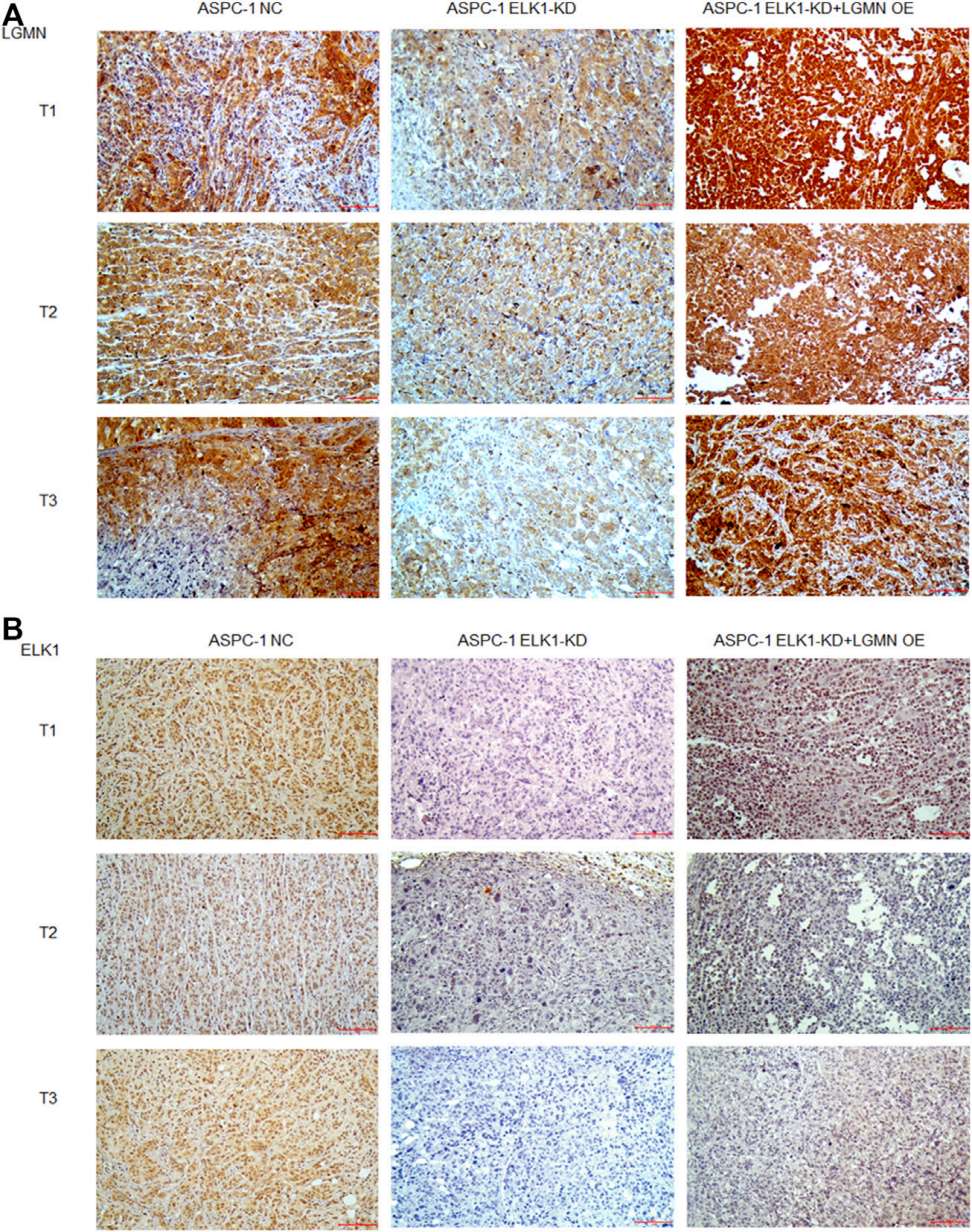
ELK1 positively correlated with LGMN expression *in vivo*. **(A**,**B)** Immunohistochemical analysis of LGMN **(A)** and ELK1 **(B)** expression of ASPC-1 cells. Figures shown that ELK1 and LGMN were expressed in ASPC-1 cells. ELK1 was not expressed in ELK1-KD group and ELK1-KD+LGMN-OE group. LGMN was rarely expressed in ELK1-KD group and rescued its expression in ELK1-KD+LGMN-OE group.

**FIGURE 8:**
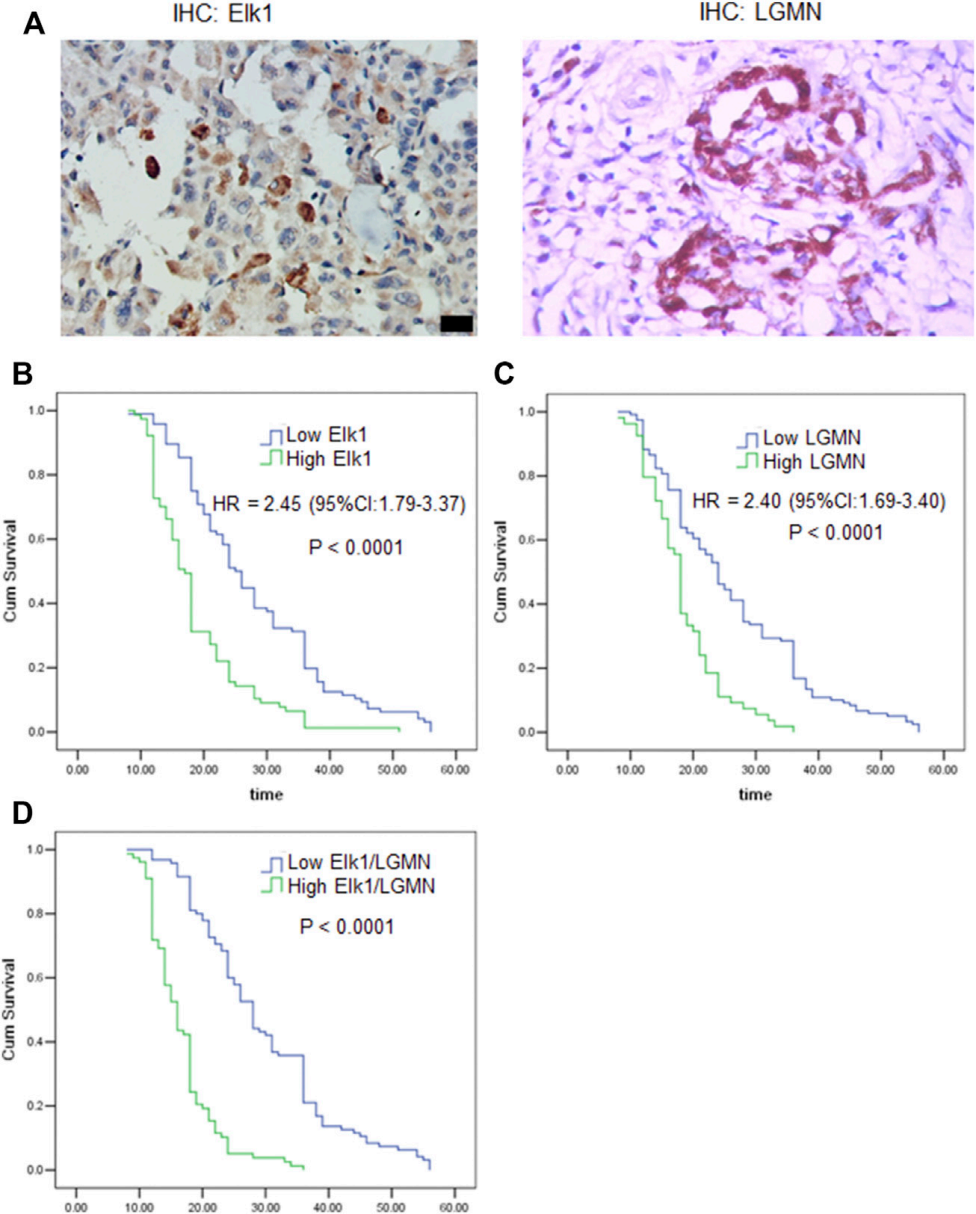
ELK1 and LGMN negatively associated with prognosis in pancreatic adenocarcinoma patients. **(A)** Expression of ELK1 and LGMN in pancreatic cancer tissues. **(B**–**D)** Kaplan-Meier survival analysis in PDAC patients (*n* = 173) according to the ELK1 and LGMN expression. High ELK1 **(B)**, LGMN **(C)** and both **(D)** expression patients showed significantly shorter 5-year OS than patients with low ELK1 **(B)**, LGMN **(C)** and both **(D)** expression in their tumors by long-rank test (*p* < 0.0001).

To evaluate the potential clinical significance of ELK1 and LGMN in the prognosis and diagnosis of PDAC, we used immunostaining to detect their expression in 176 PDAC patients. According to pathological findings, PDAC patients were divided into three groups, stage T1, T2, T3. We found that the expressions of LGMN and ELK1 were reduced in ELK1-KD group, while LGMN was saved in ELK1-KD/LGMN-OE group (Figures 7A,B). Representative expression patterns in pancreatic adenocarcinoma samples are shown in Figure 8A.

Besides, according to the expression of ELK1 and LGMN in pancreatic adenocarcinoma samples, all patients were distributed into two groups: the low expression group and the high expression group (Tables 1, 2). The immunohistochemical staining results revealed that ELK1 and LGMN levels were significantly associated with clinical stage, degree of differentiation and lymph node infiltration (Tables 1, 2).

**TABLE 2:** Association of ELK1 expression with clinicopathological variables in pancreatic ductal adenocarcinoma.

Clinicopathological characteristic	*n* (%)	Elk1 staining (*n*; %)		*p*-value
		Low	High	
Age (year)				0.137
<60	86 (48.86)	44 (51.16)	42 (48.84)	
≥60	90 (51.14)	56 (62.22)	34 (37.78)	
Sex				0.173
Male	108 (61.36)	57 (52.78)	51 (47.22)	
Female	68 (38.64)	43 (63.23)	25 (36.76)	
Clinical stages				*0.005
I/II	138 (78.41)	86 (62.32)	52 (39.86)	
III/IV	38 (21.59)	14 (36.84)	24 (63.16)	
Tumor location				0.142
Head	112 (63.64)	59 (50.89)	53 (49.11)	
Body/tail	64 (36.36)	41 (60.93)	23 (39.07)	
Tumor size				0.102
≤4 cm	58 (32.95)	38 (62.07)	20 (37.93)	
>4 cm	118 (67.05)	62 (50.85)	56 (49.15)	
Differentiation				*0.041
Weak to moderately	110 (62.50)	69 (62.73)	41 (37.27)	
Poorly	66 (37.50)	31 (46.97)	35 (53.03)	
N infiltration				0.271
Positive	165 (93.75)	92 (55.76)	73 (44.24)	
Negative	11 (6.25)	8 (72.73)	3 (27.27)	
Lymphnode infiltration				*0.050
Positive	128 (72.73)	67 (52.34)	61 (47.66)	
Negative	48 (27.27)	33 (68.75)	15 (31.25)	

To assess the relationship of ELK1 expression with patient prognosis, the log-rank test and Kaplan–Meier analysis were used to evaluate the effect of ELK1 expression on patient survival. Patients with high levels of ELK1 and LGMN expression in tumor tissues experienced significantly shorter OS than patients with low ELK1 expression (*n* = 173, *p* < 0.0001, Figures 8B–D). The mean survival time of patients with low ELK1 expression was 27.93 months (*n* = 96, 95% CI: 25.61–30.24), whereas the survival time of patients with high ELK1 expression was 18.65 months (*n* = 77, 95% CI: 16.92–20.38). The log-rank test (univariate analysis) demonstrated that patients with low LGMN expression had a longer overall survival (OS) time than patients with high LGMN expression (χ^2^ = 36.644, *p* < 0.0001). Moreover, the mean survival time of patients with low LGMN expression was 26.23 months (*n* = 119, 95% CI: 24.14–28.41), whereas the mean survival time of patients with high LGMN expression was 18.33 months (*n* = 54, 95% CI: 16.73–19.94). The log-rank test (univariate analysis) demonstrated that the patients with low LGMN expression had a longer OS time than patients with high LGMN expression (χ^2^ = 28.434, *p* < 0.0001). Multivariate Cox regression analysis was also performed to explore whether ELK1 is an independent prognostic factor for survival. As presented in Figures 8B,C, ELK1 expression and LGMN expression were identified as independent prognostic factors (ELK1: HR = 2.45, 95% CI: 1.79–3.37, *p* < 0.0001; LGMN: HR = 2.40, 95% CI: 1.69–3.40, *p* < 0.0001).

## Discussion

Pancreatic cancer is one of the most deadly cancers and and its prognosis is extremely poor, mainly due to its low early detection rate and high metastatic rate (Kamisawa et al., 2016). Though LGMN is highly expressed in the majority of human solid tumors and is associated with a more invasive and metastatic phenotype, the underlying mechanisms of its tumor-promoting effects have yet to be fully elucidated (Mai et al., 2017). We previously found that high expression of LGMN is involved in the progression of pancreatic carcinoma in an exosome-dependent manner and that LGMN independently indicated poor prognosis; however, the upstream regulation of LGMN remains unknown (Yan et al., 2018). The regulation of gene expression by transcription factors is a common cellular event. It has been reported that an inflammation-regulated transcription factor known as CCAAT-enhancer-binding protein (C/EBPβ) can modulate LGMN expression in the pathogenesis of Alzheimer’s disease (Wang et al., 2018). As a well-known tumor suppressor, the transcription factor p53 binds to intron 1 of the human LGMN gene and regulates LGMN expression at the transcriptional level (Yamane et al., 2013). In this study, we discovered that the transcription factor ELK1 functions in PDAC by regulating LGMN. Knocking down of ELK1 inhibited pancreatic cancer cells proliferation, invasion and survival, while LGMN could restore their malignancy. Overexpression of ELK1 further promoted pancreatic cancer cells proliferation, invasion and survival. An animal model showed that tumors grew slower in the ELK1-KD group and faster in the ELK1-KD/LGMN-OE group. Clinically, ELK1 and LGMN expression were positively correlated with clinical stage, degree of differentiation and lymph node infiltration. ELK1 and LGMN were determined to be independent prognostic factors for OS.

ELK1, as a transcriptional activator involved in the MAPK/ERK pathway, induces the proliferation and/or migration/invasion of bladder and prostate cancer cells as well as resistance to the cytotoxic effects of the chemotherapeutic agent cisplatin in bladder cancer cells ((Kawahara et al., 2015), (Kawahara et al., 2016)). ELK1 is a regulator of c-Fos. c-Fos has been shown to form a heterodimer with Jun, leading to the formation of the AP-1 complex and the regulation of target gene expression and is thereby involved in tumorigenesis (Angel and Karin, 1991). The direct regulation of BRCA1, variations in which are linked to increased risks of breast and ovarian cancers, by ELK1/c-Fos/Jun has also been documented (Zhong et al., 2004). Knocking down of ELK1 in MCA-exposed SVHUC-AR cells resulted in a significant decrease in the expression of several oncogenes, including c-Fos, Jun, and Myc, and a significant increase in several tumor suppressors, such as p53, PTEN, and UGT1A (Inoue et al., 2018a). Nonetheless, further search is needed to accurately determine how ELK1 signal regulates PDAC progress. In this study, we have revealed a new mechanism by which ELK1 promotes the progression of pancreatic cancer through LGMN.

Considering the unique role of ELK1 in activating gene transcription associated with cancer progression, its value as a prognostic marker deserves in-depth evaluation. Researchers have confirmed ELK1 is a strong independent predictor of prostate cancer recurrence (Pardy et al., 2020). Overexpression of phospho-ELK1, the activated form of ELK1, has also been discovered to be a predictor of poor prognosis in patients with upper urinary tract urothelial carcinoma (UUTUC) (Inoue et al., 2018b). In our study, high levels of ELK1 and LGMN were associated with advanced clinical stage and short OS, consistent with previous studies. Therefore, ELK1 can be used as a reliable indicator for predicting the prognosis of PDAC.

In conclusion, our results suggest that ELK1 promotes pancreatic cancer progression *via* LGMN and correlates with poor prognosis.

## Data Availability

The raw data supporting the conclusions of this article will be made available by the authors, without undue reservation.

## Abbreviations

AEP, asparaginyl endopeptidase; CI, confidence interval; DMEM, Dulbecco’s Modified Eagle Medium; ECL, enhanced chemiluminescence; FACS, fluorescence-activated cell sorting; FBS, fetal bovine serum; FCM, Flow cytometry; GBM, glioblastoma; LGMN, legumain; PDAC, pancreatic ductal adenocarcinoma; qRT-PCR, quantitative real-time PCR; SDS, sodium dodecyl sulfate; UUTUC, upper urinary tract urothelial carcinoma.
