# Supplementary figures and images for: ELK1 Enhances Pancreatic Cancer Progression Via LGMN and Correlates with Poor Prognosis
Source: Front Mol Biosci. 2021 Dec 13;8:764900. doi: 10.3389/fmolb.2021.764900 (PMC8711721; doi:10.3389/fmolb.2021.764900)

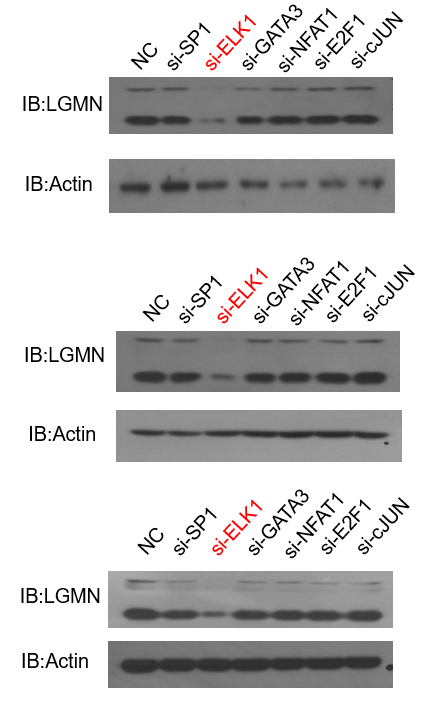

Supplement: Supplementary file 1 [file Image3.TIF]

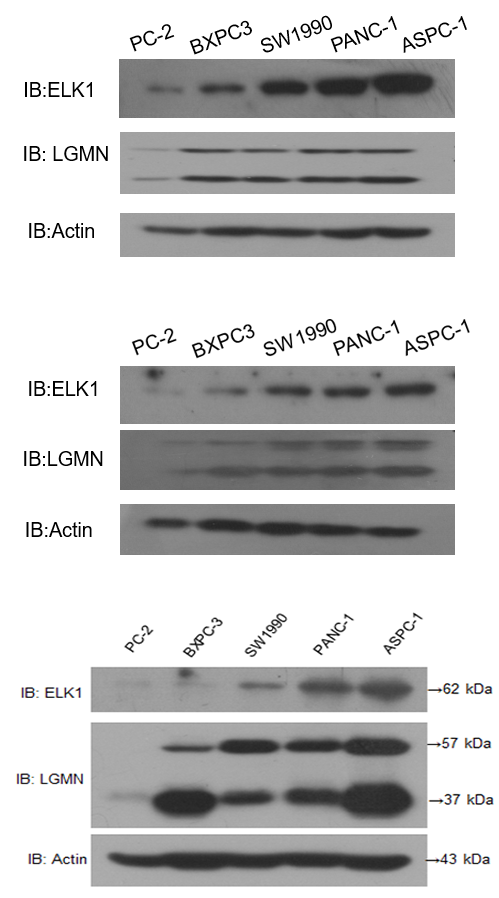

Supplement: Supplementary file 2 [file Image4.TIF]

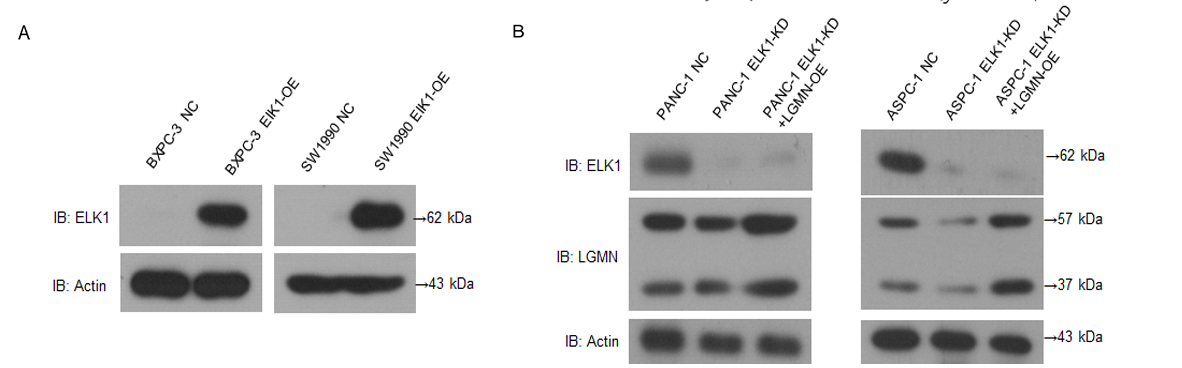

Supplement: Supplementary file 3 [file Image2.TIF]

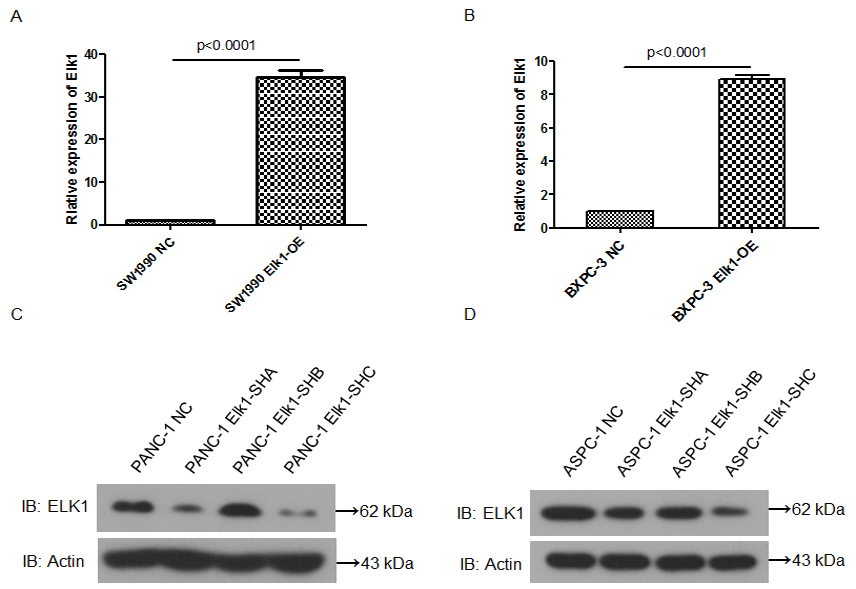

Supplement: Supplementary file 4 [file Image1.TIF]

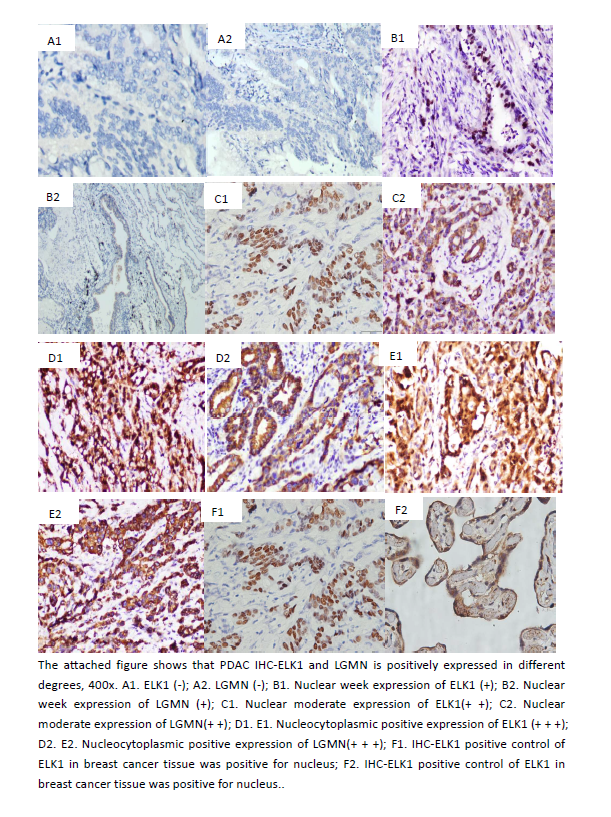

Supplement: Supplementary file 5 [file Image5.PNG]

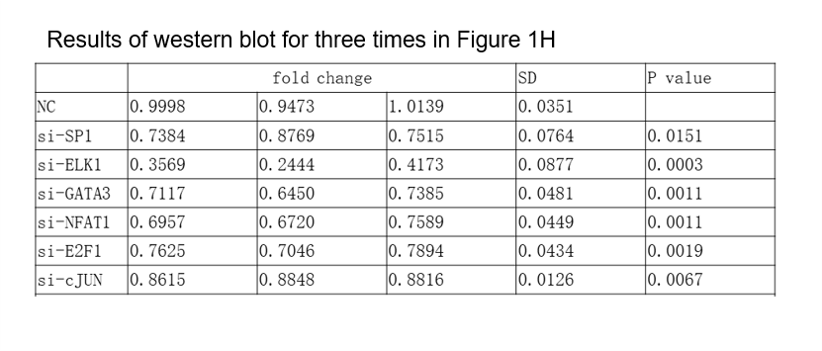

Supplement: Supplementary file 8 [file Image6.PNG]
