# Supplementary material for: ELK1 Enhances Pancreatic Cancer Progression Via LGMN and Correlates with Poor Prognosis
Source: Front Mol Biosci. 2021 Dec 13;8:764900. doi: 10.3389/fmolb.2021.764900 (PMC8711721; doi:10.3389/fmolb.2021.764900)
Supplement: Supplementary file 6 [file Table1.DOC]

Supplementary table 1. Sequences of siRNAs.

| siRNA Name | Sequences |
| --- | --- |
| si-h-ELK1_siRNA-01 | GGCAATGGCCACATCATCT |
| si-h-ELK1_siRNA-02 | GGCTACGCAAGAACAAGAC |
| si-h-ELK1_siRNA-03 | AGACCAACATGAATTACGA |
| si-h-SP1_siRNA-01 | GGTGCAAACCAACAGATTA |
| si-h-SP1_siRNA-02 | CCAACAGATTATCACAAAT |
| si-h-SP1_siRNA-03 | GCCAATAGCTACTCAACTA |
| Si-h-GATA3_siRNA-01 | CCAAGAACAGCTCGTTTAA |
| Si-h-GATA3_siRNA-02 | CCTGTGGGCTCTACTACAA |
| si-h-GATA3_siRNA-03 | GAGAAAGAGTGCCTCAAGT |
| si-h-NFAT1 (NFATC2) _siRNA-01 | GCAGAATCGTCTCTTTACA |
| si-h-NFAT1 (NFATC2) _siRNA-02 | TGCCCATGGTTGAAAGACA |
| si-h-NFAT1 (NFATC2) _siRNA-03 | GGATCTTGAAGCTTAGAAA |
| si-h-E2F1_siRNA-01 | TGGACCACCTGATGAATAT |
| si-h-E2F1_siRNA-02 | GAGAAGTCACGCTATGAGA |
| si-h-E2F1_siRNA-03 | GAGGAGTTCATCAGCCTTT |
| si-h-c_JUN_siRNA-01 | GGCACAGCTTAAACAGAAA |
| si-h-c_JUN_siRNA-02 | GGGTGCCAACTCATGCTAA |
| si-h-c_JUN_siRNA-03 | TCTACGCAAACCTCAGCAA |
