# Supplementary material for: ELK1 Enhances Pancreatic Cancer Progression Via LGMN and Correlates with Poor Prognosis
Source: Front Mol Biosci. 2021 Dec 13;8:764900. doi: 10.3389/fmolb.2021.764900 (PMC8711721; doi:10.3389/fmolb.2021.764900)
Supplement: Supplementary file 7 [file Table3.DOCX]

https://www.jianguoyun.com/p/DVN9UIYQ4p_CCRin0PYD
