# Supplementary material for: ELK1 Enhances Pancreatic Cancer Progression Via LGMN and Correlates with Poor Prognosis
Source: Front Mol Biosci. 2021 Dec 13;8:764900. doi: 10.3389/fmolb.2021.764900 (PMC8711721; doi:10.3389/fmolb.2021.764900)
Supplement: Supplementary file 9 [file Table2.DOC]

Supplementary table 2. Sequences of RT-PCR primers.

| Gene Name | Sequences |
| --- | --- |
| 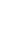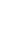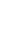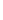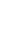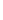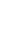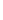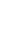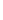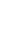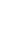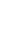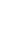human E2F1-F | ACGCTATGAGACCTCACTGAA |
| human E2F1-R | TCCTGGGTCAACCCCTCAAG |
| human SP1-F | ACGCTTCACACGTTCGGATGAG |
| human SP1-R | TGACAGGTGGTCACTCCTCATG |
| human GATA3 F | CCTACGTGCCCGAGTACAGC |
| human GATA3 R | GGTAGTGTCCCGTGCCATCT |
| 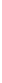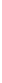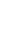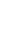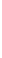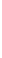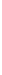human NFAT1-F | GAGCCGAATGCACATAAGGTC |
| human NFAT1-R | CCAGAGAGACTAGCAAGGGG |
| 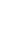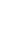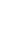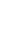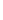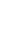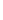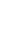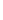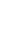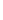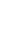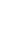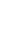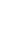human ELK1-F | CTGGACTTCACGGGATGGTG |
| human ELK1-R | CACAAACTTGTAGACGAACTTCTGG |
| human JUN-F | CCTTGAAAGCTCAGAACTCGGAG |
| human JUN-R | TGCTGCGTTAGCATGAGTTGGC |
| human RPL13a-F | CGAGGTTGGCTGGAAGTACC |
| human RPL13a-R | CTTCTCGGCCTGTTTCCGTAG |
